# Dietary intake is associated with risk of multiple myeloma and its precursor disease

**DOI:** 10.1371/journal.pone.0206047

**Published:** 2018-11-01

**Authors:** Marianna Thordardottir, Ebba K. Lindqvist, Sigrun H. Lund, Rene Costello, Debra Burton, Laufey Steingrimsdottir, Neha Korde, Sham Mailankody, Gudny Eiriksdottir, Lenore J. Launer, Vilmundur Gudnason, Tamara B. Harris, Ola Landgren, Johanna E. Torfadottir, Sigurdur Y. Kristinsson

**Affiliations:** 1 Faculty of Medicine, University of Iceland, Reykjavik, Iceland; 2 Department of Medicine, Division of Hematology, Karolinska University Hospital and Karolinska Institutet, Stockholm, Sweden; 3 Centre of Public Health Sciences, Faculty of Medicine, University of Iceland, Reykjavik, Iceland; 4 Multiple Myeloma Section, National Cancer Institute, National Institutes of Health, Bethesda, Maryland, United States of America; 5 Faculty of Food Science and Human Nutrition, University of Iceland, Reykjavik, Iceland; 6 Myeloma Service, Division of Hematologic Oncology, Memorial Sloan-Kettering Cancer Center, New York, New York, United States of America; 7 The Icelandic Heart Association, Kopavogur, Iceland; 8 Laboratory of Epidemiology and Population Sciences, Intramural Research Program, National Institute on Aging, Bethesda, Maryland, United States of America; 9 The Icelandic Cancer Society, Reykjavik, Iceland; University of South Alabama Mitchell Cancer Institute, UNITED STATES

## Abstract

The etiology of monoclonal gammopathy of undetermined significance (MGUS), the precursor state of multiple myeloma (MM), is mostly unknown and no studies have been conducted on the effect of diet on MGUS or progression from MGUS to MM. We aimed to explore the association between common foods and MGUS and progression to MM. Data from the population-based AGES Study (N = 5,764) were utilized. Food frequency questionnaire was used to assess dietary intake during adolescence, midlife, and late life. Serum protein electrophoresis and serum free light-chain assay was performed to identify MGUS (n = 300) and LC-MGUS cases (n = 275). We cross linked our data with the Icelandic Cancer Registry to find cases of MM in the study group. We found that intake of fruit at least three times per week during adolescence was associated with lower risk of MGUS when compared to lower fruit consumption (OR = 0.62, 95% CI 0.41–0.95). We additionally found that intake of fruit at least three times per week during the late life period was associated with decreased risk of progressing from MGUS to MM (HR = 0.34, 95% CI 0.13–0.89) when compared to lower intake. Adolescent intake of fruit may reduce risk of MGUS, whereas fruit intake after MGUS onset may reduce risk of progressing to MM. Our findings suggest that diet might alter the risk of developing MGUS and progression to MM.

## Introduction

All cases of the plasma cell malignancy multiple myeloma (MM) are preceded by monoclonal gammopathy of undetermined significance (MGUS) [1, 2], a premalignant asymptomatic condition characterized by the presence of an M-protein in serum or by abnormal ratio between the free light-chains kappa and lambda (light-chain MGUS), without indication of MM or other lymphoproliferative (LP) diseases [3–5]. The prevalence of MGUS is approximately 5% in those older than 70 years and increases with age [6]. It is estimated that average risk of progression from MGUS to MM is approximately 1% per year [7, 8]. Light-chain MGUS (LC-MGUS) has been described as a precursor to light-chain MM, with a prevalence of 0.7–0.8% [4, 5].

As previously reported, the etiology of MGUS and LC-MGUS is mostly unknown [9]. However, studies have reported a higher risk of MGUS among males [6], black race [10, 11], in individuals with family history of MGUS and related diseases [12], in individuals with previous personal or family history of immune-related conditions [13, 14], and recently in those who have been largely exposed to Agent Orange, an herbicide and defoliant chemical [15]. The literature on the etiology of MM is more extensive. An elevated risk of MM has been found to be associated with low occupation-based socioeconomic status, income, education [16], and high body mass index (BMI) [9, 17, 18]. The International Agency for Research on Cancer has recently concluded, that there is now adequate evidence behind the association between body weight and MM [19]. We have recently shown that high BMI, measured during midlife (≈50 years old), was associated with an increased risk of progressing from MGUS to MM and other LP diseases later in life, suggesting that exposures that originate during the midlife period, and perhaps earlier in life, play a role in the pathogenesis of MM and related diseases diagnosed later in life [9]. Since obesity has various underlying causes this indicates that lifestyle-related factors, such as diet, are important risk factors for MM. However, epidemiological evidence on the effect of diet on MM is scarce and the results are inconclusive [20–24]. The strongest evidence exists for fish intake, where inverse association has been reported in few case-control studies [21, 24–26], two of which reported dose-response relationship [21, 25]. No studies, to our knowledge, on the association between diet and MGUS or LC-MGUS have been conducted. Additionally, MGUS has been detected in individuals as young as 10–19 years old [27], emphasizing the importance of studying the effect of early and midlife exposures on MGUS. Many of the residential regions in Iceland were relatively isolated during the first half of the 20^th^ century. Due to that, there was a considerable variation in diet across the country. The inhabitants’ diets was largely limited to locally produced food available on side such as fish in sea villages and livestock at the farm [28]. Availability of fruit and vegetables was limited due to unfavorable weather conditions and limited import. Iceland is therefore an ideal forum for dietary research due to extremes in intake of common food groups. During the second half of the 20^th^ century the diet became more westernized [29].

As all MM cases first go through the MGUS stage it is important to disentangle these associations with the objective to identify separate risk factors for MGUS, LC-MGUS, and progression to MM. The overarching aim of this study was therefore to analyze the association between diet throughout the lifespan and risk of MGUS and LC-MGUS using population-based data. Secondly, we aimed to analyze whether diet was a risk factor for progression to MM and other LP diseases in individuals with MGUS or LC-MGUS.

## Materials and methods

### Study population

For this study we used data from the Age, Gene/Environment Susceptibility–Reykjavik Study (AGES Study). The AGES Study is a continuation of the population-based Reykjavik Study, initiated in 1967 when all residents, born 1907–1935, of the Reykjavik metropolitan area were invited to participate in a prospective cohort study with the objective of examining risk factors for cardiovascular diseases. A total of 71% (n = 19,350) of the invited residents consented to participate during the years of 1967–1996 [30–32]. Of the 11,549 Reykjavik Study cohort members still alive in March 2002, when the AGES Study was initiated, 8,030 individuals were randomly chosen to take part in the study. By 2006, when the study ended, 5,764 (71.8%) had participated. Detailed description of the study and collection of data has been previously published [32], however in short, data were collected during three separate examinations using standardized protocols. The first visit included e.g. blood draw, anthropometry, electrocardiography, and extensive questionnaire including e.g. health history, lifestyle practices, and food history. The second examination included imaging protocols and the third examination included e.g. dementia assessments and vision screening [32].

At study entry the participants signed an informed consent form. The study was approved by the Icelandic Data Protection Authority, the Icelandic National Bioethics Committee (VSN-00-063-V35), and the Institutional Review Board of the National Institute on Aging in the USA.

### Dietary habits across the lifespan

At AGES Study entry the participants provided retrospective information on dietary habits during adolescence (14–19 years old) and midlife (40–50 years old), as well as information on current dietary habits using a food frequency questionnaire. The questionnaire included a total of 63 questions (16 from the adolescent period, 17 from the midlife period, and 30 from the late life period) regarding intake of common foods and food groups i.e. total fish intake (and additional question on salted or smoked fish), fish oil, total meat intake (and additional question on salted or smoked meat), milk and milk products, fruit, vegetables (excluding potatoes), rye bread and flatbread, blood or liver sausage, oatmeal and muesli, potatoes, and whole wheat bread. Only foods and food groups that were included in the questionnaire from all three life stages were used for this study, except for whole wheat bread (midlife and late life only). The participants reported frequency of intake in each time period using the following response categories for meat, milk and milk products, fruit, vegetables, rye bread and flatbread, blood or liver sausage, oatmeal and muesli, potatoes, and whole wheat bread: *never*, *less than once a week*, *1–2 times a week*, *3–4 times a week*, *5–6 times a week*, *daily*, and *more than once a day*. For fish oil the categories were the same except for: *more than once a day*. The categories for salted and smoked meat and fish were: *never*, *less than once a month*, *1–3 times a month*, *1–2 times a week*, *3–6 times a week*, and *daily or more often*. The food frequency questionnaire included three questions on fish consumption; frequency of consumption of fish in salad or as topping on bread, fish as main meal (including salted or smoked fish), and consumption of salted or smoked fish separately. Weekly intake of fish meals and fish in salad or as topping was combined into one variable and the daily total intake was converted into total fish portions per week, as has been previously described [33]. The validation study of the midlife diet took advantage of available data from a detailed nutrition study performed 18 years previously, using dietary history and the present diet was validated using a 3-day dietary history as a reference method. The AGES food frequency questionnaire was found suitable to rank individuals by their intake for most food groups from the midlife and late period [34, 35].

### Ascertainment of outcomes

As previously described [9, 36], a conventional agarose-gel serum protein electrophoresis (SPEP) was performed in 2013–2014 on all subjects from the AGES Study cohort to identify MGUS cases. A 0.5 mL serum sample, collected at study entry (2002–2006), was obtained for each study subject and samples with an equivocal or definite M-protein present on SPEP were then subjected to serum protein immunofixation for conformation and typing of the M-protein [6]. Serum free light chain (FLC) assay was performed on all samples [37]. The sensitivity and specificity of the laboratory tests have been previously published [38]. All testing was done by individuals blinded to all demographics and other details related to the samples.

MGUS cases were defined as having M-protein bands (detectable on SPEP or immunofixation) and an elevated M-protein concentration (≤30g/L) [39]. LC-MGUS cases were defined as having no visible M-protein, a pathological FLC ratio (<0.26 or >1.65) on FLC analysis, and an increased free light-chain concentration (f-kappa >19.4 mg/L, f-lambda >26.3 mg/L) [40]. We ascertained MM diagnosis and diagnosis of other LP diseases through linkage with the nationwide Icelandic Cancer Registry [41]. The start date of follow-up was at AGES Study entry (March 2002-February 2006) until March 2014.

### Exclusion from analysis

For this study we excluded a total of 40 (0.7%) subjects from analysis, 21 due to previous LP diseases, 16 due to missing blood sample, one due to absent consent form, and one subject had high M-protein concentration at baseline and therefore fulfilled criteria for smoldering MM. One subject was additionally excluded from the progression analysis due to lack of follow-up. Our analyses include individuals responding to the dietary questions, ranging from 5,270 to 5,304 from the adolescent period, from 5,279 to 5,301 from the midlife period, and from 507–511 from the late life period, depending on the question.

### Statistical analysis

A total of 12 food items were analyzed for the adolescent period and 13 for both midlife and the late life period. Logistic regression was used to estimate odds ratios (OR) and 95% confidence intervals (95% CI) for MGUS and LC-MGUS according to adolescent and midlife diet. For each type of food or food group, participants were grouped according to their frequency of intake or portions per week (total fish consumption only). Each type of food or food group was analyzed in an age and sex adjusted model (Model 1) and additionally in a fully adjusted model where all the foods and food groups were simultaneously added to one model, including age and sex (Model 2). An additional adjustment was made for physical activity and BMI measured in midlife in the adolescent and midlife models, and since the adjustment did not affect our results physical activity and BMI were omitted from the models. Additionally, we cross-classified the intake from the adolescent and midlife periods by combining individuals with low adolescent intake and low midlife intake for each type of food and set them as a reference group for low and high, high and low, and high and high intake at these time periods. We then used logistic regression to test the association between the cross-classified categories of intake and MGUS and LC-MGUS combined. Models were adjusted for age and sex.

Cox proportional hazard regression was used to test whether diet at study entry was a associated with progression from MGUS/LC-MGUS to MM or other LP diseases. For this analysis only individuals with MGUS and LC-MGUS were used, and due to few cases of MM and other LP disease they were not analyzed separately. Results are presented as hazard ratios (HR) with 95% CI. All models were adjusted for sex and age at AGES Study entry. We further adjusted our models for BMI and physical activity and we tested the association in a model where all the foods were simultaneously added to one model. Due to few number of cases and therefore low statistical power, and no effect on results, these analyses were not included in our results. All analyses were performed in R version 3.3.3.[42]

### Sensitivity analysis

As described in detail previously [9, 36], we had an unusually high prevalence of LC-MGUS in our cohort (4.8%), mainly due to a high prevalence of kappa cases (96%). The distribution of log-transformed kappa and lambda values was found to resembled the normal distribution, and therefore the cut-off for the involved chains was moved to the 97.5^th^ percentile. A definition of LC-MGUS as a pathological FLC-ratio of < 0.26 and > 1.65, in combination with an increased concentration of more than 40.0 mg/L of the light-chain involved was used to perform sensitivity analysis on the association between diet and MGUS/LC-MGUS.

## Results

The mean age of participants was 77 years (range 66–98) at study baseline. MGUS and LC-MGUS was identified in 300 (5.2%) and 275 (4.8%) subjects, respectively (Table 1). Using the modified definition of LC-MGUS resulted in 52 cases. By cross linking the AGES Study cohort to the Icelandic Cancer Registry we found that 18 participants progressed to MM, of which one from LC-MGUS during a median follow-up of 8 years. Additionally, 11 progressed to other LP diseases (Hodgkin’s, Non-Hodgkin’s lymphoma, Waldenström's macroglobulinemia, lymphoid leukemia, chronic lymphocytic leukemia, and acute lymphocytic leukemia), of which two from LC-MGUS.

**Table 1:** Characteristics of study participants at AGES study entry.

	Without MGUS	MGUS	LC-MGUS	MM	LP
	n = 5,150 (90.0%)	n = 300 (5.2%)	n = 275 (4.8%)	n = 18 (3.1%)*	n = 11 (5.1%)*
Gender, n. (%)					
Female	3,046 (59.1)	141 (47.0)	119 (43.3)	10 (55.6)	13 (46.4)
Male	2,104 (40.9)	159 (53.0)	156 (56.7)	8 (44.4)	15 (53.6)
Mean age, years (range)	76.8 (66–98)	78.3 (67–93)	79.4 (66–97)	77.8 (69–87)	77.5 (68–87)
BMI, kg/m^2^ (mean)	27.0	26.7	27.0	27.2	26.5
BMI midlife, kg/m^2^ (mean)	25.2	25.5	25.6	26.2	26.2

*Proportion of cases that progressed from MGUS or LC-MGUS.

Abbreviations: MGUS—Monoclonal gammopathy of undetermined significance, LC-MGUS—Light chain monoclonal gammopathy of undetermined significance, MM—Multiple myeloma, LP—Other lymphoproliferative diseases.

### Adolescent intake

We found, in Model 2, that intake of fruit at least three times per week during the adolescent period was associated with lower risk of MGUS when compared intake less than three times per week (OR = 0.62, 95% CI 0.41–0.95). Consumption of other food items during that period was not associated with MGUS (Table 2). Additionally, we found that intake of fish at least two times per week was associated with higher risk of LC-MGUS (OR = 1.33, 95% CI 1.02–1.73) when compared to intake less than two times per week. Adolescent consumption of other foods was not associated with LC-MGUS (Table 2). In our sensitivity analysis we did not find any association between fish intake and risk of LC-MGUS in Model 2 as we did in our primary analysis. No association was found between consumption of other food items and LC-MGUS in our sensitivity analysis.

**Table 2:** The association between diet in adolescence and MGUS and LC-MGUS.

				Model 1^a^				Model 2^b^			
	No MGUS	MGUS	LC-MGUS	MGUS		LC-MGUS		MGUS		LC-MGUS	
	n (%)	n (%)	n (%)	OR	95%CI	OR	95% CI	OR	95%CI	OR	95% CI
**Fish**											
≤ 2 portions p/w	2,335 (49.1)	140 (52.2)	99 (40.7)	1.00		1.00		1.00		1.00	
> 2 portions p/w	2,424 (50.9)	128 (47.8)	144 (59.3)	0.85	0.66–1.09	1.33	1.02–1.73	0.88	0.68–1.14	1.39	1.06–1.83
**Fish oil**											
less than weekly	2,280 (47.6)	119 (44.4)	128 (52.7)	1.00		1.00		1.00		1.00	
weekly or more	2,509 (52.4)	149 (55.6)	115 (47.3)	1.13	0.88–1.45	0.82	0.63–1.06	1.14	0.89–1.48	0.87	0.66–1.14
**Salted fish**											
3 times a month or less	2,210 (46.4)	128 (47.8)	114 (46.9)	1.00		1.00		1.00		1.00	
once p/w or more	2,555 (53.6)	140 (52.2)	129 (53.1)	0.85	0.66–1.10	0.84	0.64–1.09	0.95	0.71–1.26	0.89	0.66–1.21
**Meat**											
2 times p/w or less	1,689 (35.3)	103 (38.6)	91 (37.6)	1.00		1.00		1.00		1.00	
3 times p/w or more	3,093 (64.7)	164 (61.4)	151 (62.4)	0.87	0.67–1.12	0.89	0.68–1.17	0.89	0.68–1.15	0.86	0.62–1.14
**Smoked/salted meat**											
3 times a month or less	3,111 (65.2)	184 (68.7)	157 (64.9)	1.00		1.00		1.00		1.00	
Once a week or more	1,663 (34.8)	84 (31.3)	85 (35.1)	0.79	0.60–1.03	0.90	0.68–1.18	0.82	0.61–1.10	0.94	0.69–1.28
**Milk and milk products**											
less than daily	1,074 (22.4)	65 (24.3)	63 (25.9)	1.00		1.00		1.00		1.00	
daily	3,719 (77.6)	203 (75.7)	180 (74.1)	0.87	0.65–1.17	0.79	0.59–1.07	0.91	0.67–1.24	0.85	0.62–1.17
**Fruit**											
2 times p/w or less	4,077 (85.3)	241 (89.9)	217 (89.7)	1.00		1.00		1.00		1.00	
3 times p/w or more	701 (14.7)	27 (10.1)	25 (10.3)	0.71	0.47–1.06	0.75	0.49–1.15	0.62	0.41–0.95	0.81	0.52–1.26
**Vegetables**											
2 times p/w or less	3,517 (73.5)	197 (73.8)	197 (81.1)	1.00		1.00		1.00		1.00	
3 times p/w or more	1,265 (26.5)	70 (26.2)	46 (18.9)	1.06	0.80–1.41	0.71	0.51–0.99	1.17	0.87–1.57	0.78	0.55–1.10
**Rye bread/flatbread**											
less than daily	2,481 (52.0)	141 (52.6)	120 (49.4)	1.00		1.00		1.00		1.00	
daily	2,289 (48.0)	127 (47.4)	123 (50.6)	0.88	0.68–1.14	0.94	0.72–1.22	0.94	0.71–1.23	1.00	0.75–1.34
**Sausage/liver**											
less than weekly	1,252 (26.1)	74 (27.6)	68 (28.0)	1.00		1.00		1.00		1.00	
weekly or more	3,539 (73.9)	194 (72.4)	175 (72.0)	0.84	0.63–1.11	0.78	0.58–1.04	0.86	0.65–1.18	0.85	0.61–1.18
**Oatmeal/muesli**											
2 times p/w or less	2,119 (44.4)	110 (41.4)	108 (44.6)	1.00		1.00		1.00		1.00	
3 times p/w or more	2,650 (55.7)	156 (58.6)	134 (55.4)	1.02	0.79–1.31	0.84	0.64–1.09	1.09	0.83–1.44	0.93	0.70–1.24
**Potatoes**											
less than daily	483 (10.1)	35 (13.1)	25 (10.3)	1.00		1.00		1.00		1.00	
daily	4,300 (89.9)	233 (86.9)	217 (89.7)	0.76	0.52–1.10	0.99	0.64–1.51	0.81	0.55–1.20	1.04	0.67–1.63

^a^Adjusted for age and sex.

^b^Adjusted for age, sex and the other food groups.

Abbreviations: MGUS—Monoclonal gammopathy of undetermined significance, LC-MGUS—Light chain monoclonal gammopathy of undetermined significance, OR—Odds ratio, CI—confidence interval

### Midlife intake

We found in Model 2 that intake of whole wheat bread at least five times per week was associated with lower risk of MGUS (OR = 0.75, 95% CI 0.57–0.99) when compared to participants with intake of less than five times per week. Intake of other foods from midlife was not associated with MGUS (Table 3). Midlife intake of the tested food items was not associated with LC-MGUS (Table 3). In our sensitivity analysis we found in Model 2 that midlife intake of meat at least three times per week and daily intake of rye bread and flatbread was associated with lower risk of LC-MGUS (OR = 0.44, 95% CI 0.23–0.84 and OR = 0.32, 95% CI 0.14–0.78, respectively) when compared to lower intake. No association was found between consumption of other food items from the midlife periods and LC-MGUS in our sensitivity analysis.

**Table 3:** The association between midlife diet and MGUS and LC-MGUS.

				Model 1^a^				Model 2^b^			
	No MGUS	MGUS	LC-MGUS	MGUS		LC-MGUS		MGUS		LC-MGUS	
	n (%)	n (%)	n (%)	OR	95%CI	OR	95% CI	OR	95%CI	OR	95% CI
**Fish**											
≤ 2 portions p/w	565 (11.8)	35 (13.1)	33 (13.6)	1.00		1.00		1.00		1.00	
> 2 portions p/w	4,204 (88.2)	233 (86.9)	209 (86.4)	0.84	0.58–1.22	0.77	0.52–1.13	0.87	0.59–1.28	0.80	0.54–1.19
**Fish oil**											
less than weekly	1,852 (38.7)	97 (36.3)	97 (39.9)	1.00		1.00		1.00		1.00	
weekly or more	2,931 (61.3)	170 (63.7)	146 (60.1)	1.09	0.84–1.41	0.94	0.72–1.23	1.10	0.84–1.44	0.95	0.72–1.25
**Salted fish**											
3 times a month or less	3,241 (67.8)	187 (69.8)	161 (66.3)	1.00		1.00		1.00		1.00	
once p/w or more	1,541 (32.2)	81 (30.2)	82 (33.7)	0.81	0.62–1.07	0.91	0.67–1.20	0.82	0.60–1.13	0.90	0.65–1.25
**Meat**											
2 times p/w or less	1,809 (37.8)	106 (39.6)	93 (38.3)	1.00		1.00		1.00		1.00	
3 times p/w or more	2,977 (62.2)	162 (60.4)	150 (61.7)	0.94	0.73–1.21	1.03	0.78–1.35	0.93	0.72–1.21	1.00	0.76–1.32
**Smoked/salted meat**											
3 times a month or less	3,530 (73.8)	199 (74.3)	170 (70.2)	1.00		1.00		1.00		1.00	
Once a week or more	1,252 (26.2)	69 (25.7)	72 (29.8)	0.89	0.67–1.18	1.05	0.79–1.40	0.95	0.68–1.32	1.12	0.80–1.56
**Milk and milk products**											
less than daily	1,957 (40.9)	99 (37.1)	90 (37.2)	1.00		1.00		1.00		1.00	
daily	2,823 (59.1)	168 (62.9)	152 (62.8)	1.11	0.86–1.43	1.08	0.82–1.41	1.17	0.89–1.53	1.15	0.87–1.53
**Fruit**											
2 times p/w or less	3,271 (68.4)	185 (69.0)	181 (74.5)	1.00		1.00		1.00		1.00	
3 times p/w or more	1,512 (31.6)	83 (31.0)	62 (25.5)	1.10	0.84–1.44	0.88	0.65–1.19	1.19	0.87–1.62	0.84	0.60–1.18
**Vegetables**											
2 times p/w or less	3,081 (64.6)	182 (68.2)	162 (66.7)	1.00		1.00		1.00		1.00	
3 times p/w or more	1,692 (35.4)	85 (31.8)	81 (33.3)	0.92	0.70–1.20	1.02	0.77–1.34	0.84	0.62–1.14	1.11	0.81–1.52
**Rye bread/flatbread**											
less than daily	3,264 (68.1)	185 (69.0)	166 (68.3)	1.00		1.00		1.00		1.00	
daily	1,526 (31.9)	83 (31.0)	77 (31.7)	0.86	0.66–1.13	0.83	0.62-.1.10	0.91	0.68–1.22	0.84	0.62–1.15
**Sausage/liver**											
less than weekly	2,322 (48.5)	123 (45.9)	112 (46.1)	1.00		1.00		1.00		1.00	
weekly or more	2,466 (51.5)	145 (54.1)	131 (53.9)	1.05	0.82–1.35	1.00	0.77–1.30	1.07	0.82–1.39	1.00	0.76–1.33
**Oatmeal/muesli**											
2 times p/w or less	2,910 (60.9)	147 (55.1)	140 (57.9)	1.00		1.00		1.00		1.00	
3 times p/w or more	1,869 (39.1)	120 (44.9)	102 (42.1)	1.18	0.92–1.52	1.01	0.77–1.31	1.27	0.94–1.60	1.05	0.80–1.40
**Potatoes**											
less than daily	742 (15.5)	48 (17.9)	40 (16.5)	1.00		1.00		1.00		1.00	
daily	4,046 (84.5)	220 (82.1)	203 (83.5)	0.80	0.58–1.11	0.86	0.60–1.22	0.82	0.58–1.16	0.88	0.61–1.28
**Whole wheat bread**											
4 times p/w or less	1,489 (31.2)	101 (38.1)	87 (36.1)	1.00		1.00		1.00		1.00	
5 times p/w or more	3,291 (68.8)	164 (61.9)	154 (63.9)	0.76	0.59–0.98	0.83	0.64–1.09	0.75	0.57–0.99	0.86	0.64–1.15

^a^Adjusted for age and sex.

^b^Adjusted for age, sex and the other food groups.

Abbreviations: MGUS—Monoclonal gammopathy of undetermined significance, LC-MGUS—Light chain monoclonal gammopathy of undetermined significance, OR—Odds ratio, CI—confidence interval.

### Cross-classification across two time points

Looking at both the adolescent and midlife period together, no association was found between any of the food items and MGUS/LC-MGUS (S1 Table). However, in our sensitivity analysis (S2 Table) we found that daily intake of rye bread and flatbread and potatoes during both the adolescent and midlife periods was associated with lower risk of MGUS/LC-MGUS when compared to less than daily intake at both periods (OR = 0.70, 95% CI 0.55–0.95 and OR = 0.63, 95% CI 0.45–0.96, respectively).

### Progression to multiple myeloma and other lymphoproliferative diseases

We found, in a sex and age adjusted model, that fruit intake at least three times per week was inversely associated with risk of progression to MM (HR = 0.34, 95% CI 0.13–0.89) when compared to intake less than three times per week. The association remained statistically significant when cases of other LP diseases were combined with MM cases (HR = 0.45, 95% CI 0.21–0.96). Intake of other food items was not associated with risk of progression to MM and other LP diseases (Table 4). Similar results were found when analyzing risk of progression in MGUS cases only.

**Table 4:** The association between late life diet and risk of progression from MGUS and LC-MGUS to multiple myeloma and other lymphoproliferative diseases.

	Total	Multiple myeloma	Multiple myeloma and other LP	Multiple myeloma *		Multiple myeloma and other LP*	
	n (%)	n (%)	n (%)	HR	95%CI	HR	95% CI
**Fish**							
≤ 2 portions p/w	149 (29.2)	7 (41.2)	10 (35.7)	1.00		1.00	
> 2 portions p/w	361 (70.8)	10 (58.8)	18 (64.3)	0.62	0.24–1.64	0.77	0.35–1.67
**Fish oil**							
less than weekly	152 (29.9)	6 (35.3)	9 (32.1)	1.00		1.00	
weekly or more	357 (70.1)	11 (64.7)	19 (64.9)	0.81	0.30–2.22	0.91	0.41–2.03
**Salted fish**							
less than once a month	371 (72.7)	13 (76.5)	21 (75.0)	1.00		1.00	
once a month or more	139 (27.3)	4 (23.5)	7 (25.0)	0.95	0.30–2.95	1.00	0.42–2.39
**Meat**							
2 times p/w or less	186 (36.4)	5 (29.4)	6 (21.4)	1.00		1.00	
3 times p/w or more	325 (63.6)	12 (70.6)	22 (78.6)	1.47	0.51–4.21	2.18	0.88–5.41
**Smoked/salted meat**							
less than once a month	341 (66.7)	14 (82.4)	20 (71.4)	1.00		1.00	
once a month or more	170 (33.3)	3 (17.6)	8 (28.6)	0.47	0.13–1.68	0.84	0.36–1.96
**Milk and milk products**							
less than daily	223 (43.7)	8 (47.1)	10 (35.7)	1.00		1.00	
daily	287 (56.3)	9 (52.9)	18 (64.3)	0.96	0.37–2.50	1.56	0.72–3.39
**Fruits**							
2 times p/w or less	151 (29.5)	9 (52.9)	13 (46.4)	1.00		1.00	
3 times p/w or more	360 (70.5)	8 (47.1)	15 (53.6)	0.34	0.13–0.89	0.45	0.21–0.96
**Vegetables**							
2 times p/w or less	270 (52.9)	11 (64.7)	16 (57.1)	1.00		1.00	
3 times p/w or more	240 (47.1)	6 (35.3)	12 (42.9)	0.54	0.20–1.49	0.78	0.36–1.67
**Rye bread/flatbread**							
2 times p/w or less	225 (44.4)	10 (58.8)	15 (53.6)	1.00		1.00	
3 times p/w or more	282 (55.6)	7 (41.2)	13 (46.4)	0.57	0.22–1.52	0.70	0.33–1.48
**Sausage/liver**							
never	111 (21.8)	3 (17.6)	6 (21.4)	1.00		1.00	
ever	398 (78.2)	14 (82.4)	22 (78.6)	1.40	0.40–4.88	1.07	0.43–2.64
**Oatmeal/muesli**							
2 times p/w or less	265 (51.9)	9 (52.9)	17 (60.7)	1.00		1.00	
3 times p/w or more	246 (48.1)	8 (47.1)	11 (39.3)	0.96	0.36–2.51	0.69	0.32–1.50
**Potatoes**							
less than daily	220 (43.1)	7 (41.2)	12 (42.9)	1.00		1.00	
daily	290 (56.9)	10 (58.8)	16 (57.1)	1.12	0.42–2.96	1.04	0.49–2.20
**Whole wheat bread**							
4 times p/w or less	130 (25.5)	4 (23.5)	4 (14.3)	1.00		1.00	
5 times p/w or more	380 (74.5)	13 (76.5)	24 (85.7)	1.02	0.33–3.15	1.93	0.66–5.56

*Models adjusted for age and sex.

Abbreviations: MGUS—Monoclonal gammopathy of undetermined significance, LC-MGUS–Light chain monoclonal gammopathy of undetermined significance, LP–lymphoproliferative, OR—Odds ratio, CI—confidence interval.

## Discussion

In this population-based study we found, using a food frequency questionnaire for evaluation of dietary intake during three separate time periods, that food intake may affect risk of MGUS, and specifically that intake of fruit at least three times per week during adolescence and midlife intake of whole wheat bread at least five times per week, was associated with lower risk of MGUS when compared to lower intakes. Additionally, we found, albeit based on few cases, that late life intake of fruit at least three times per week in individuals with MGUS or LC-MGUS was associated with lower risk of progressing to MM and other LP diseases. Adjusting for BMI and physical activity did not change the results. These findings suggest that dietary habits might influence the etiology of MGUS and progression to MM and other LP diseases.

We found that intake of fruit at least three times per week during the adolescent period was inversely associated with risk of MGUS later in life when compared to lower intake, and, based on a small number of cases, we found that late life intake of fruit at least three times per week in patients with MGUS or LC-MGUS was associated with lower risk of progression. The findings are in accordance with other studies consistently showing high intake of fruit and other plant foods to be inversely associated with different types of cancer [43, 44]. A review of findings from the European Prospective Investigation into Cancer and Nutrition (EPIC) from 2014 reported an inverse association between fruit intake and cancer risk at some sites [43]. The literature regarding fruit and risk of MM is scarce. To date, only two studies have examined the role of fruit in the etiology of MM. Both are US-based case-control studies on adults already diagnosed with MM, with no association reported [21, 23]. Major differences in the amount (intake levels) and time of exposure (period of life) and time of assessment (exposure assessed after MM diagnosis) could explain the discrepancy with regards to our study. A potential biologic mechanism for our finding is perhaps the anti-carcinogenic effect of vitamin C as it traps free radicals and protects against oxidation [45]. Nevertheless, very few participants reported daily or more than daily fruit intake in present study. Therefore, the cut-off for high intake of fruit in our study was three times per week or more. This would normally be considered low intake, but due to the low frequency of fruit consumption the cut-off could not be higher. The beneficial threshold for both risk of MGUS and progression to MM may therefore be low. Since access to fruit was limited in Iceland, especially during the adolescent period for this population and in rural areas, it is possible that fruit intake was an indicator of higher social status or overall healthier life-style, and therefore the higher intake individuals were at lower risk for multiple health outcomes, including MGUS and MM.

Interestingly, we did not find an association between intakes of fish and fish liver oil and MGUS or progression to MM. Although not significant, the point estimates in the progression analysis were in the same direction as previous studies on the association between fish intake and risk of MM have reported. A few case-control studies have reported an inverse association between fish intake and risk of MM [21, 24–26], including two that have found a dose-response relationship [21, 25]. Both of these studies report much lower fish intake than reported in our study. A suggested mechanism for the association is the cancer preventive effect of the polyunsaturated omega-3 essential fatty acid (n-3) [46]. Fish intake is a common indicator for social status [47]. However, for the Icelandic population, fish was widely available and the diet was characterized by a very high fish intake. Due to the high intake we do not have a non-exposed reference group in our fish intake analysis. Although our population has a uniquely high intake of fish, the most common types were lean fish, low in n-3. Nevertheless, the Icelandic population has high levels of the marine derived n-3 in both diet and plasma, even those with low intake of fish liver oil, a common supplement in Iceland, rich in n-3.[48] Therefore, we cannot rule out the option that a possible beneficial threshold of n-3 might already have been reached by our low intake fish and fish liver oil reference groups. Another reason for not finding an association between fish consumption and MM in our study could be the lack of a reference group in the study that did not consume fish.

We found that intake of whole wheat bread at least five time per week was inversely associated with MGUS when compared to lower intake. To date, only one study has analyzed the association between whole grain intake and risk of MM. A case-control study based on 120 MM cases below the age of 75 years reported that high intake of whole grain foods was inversely associated with MM risk in women (OR = 0.5, p ≤ 0.05) but not in men [49]. Potential mechanisms that could mediate the effect of whole grains on MM risk have not been sufficiently explored. However, whole grains have been suggested to have positive effects on long-term insulin secretion [50] which could be of importance regarding MM since increased availability of IGF-1 can increase MM cell proliferation and prevent apoptosis [51].

We found inconsistency in LC-MGUS risk between our primary and sensitivity analyses when analyzing associations with fish, meat, and rye bread and flatbread intake and additionally when analyzing the association between rye bread and flatbread and potatoes and MGUS (MGUS and LC-combined) in our cross-classification analysis. The prevalence of LC-MGUS was 4.8% in our cohort, which is considerably higher than has been noted in previous studies [4, 5]. Although this is an elderly population the difference is substantial. It is therefore difficult to draw conclusions from the results.

The strength of our study is the well-established population-based cohort design with limited threat to both internal and external validity. Another major strength is the ability to study dietary intake throughout the lifespan, as studying early life exposures is challenging yet also important since many diseases originate early in life. Few studies have been able to provide data on adolescent or midlife diet combined with detailed ascertainment of later life health outcomes and we believe our study is unique in that aspect. Additionally, a major strength is the utilization of a validated food frequency questionnaire since majority of the questions had an acceptable correlation and the questionnaire was found suitable to rank individuals by their intake for most food groups when compared to a reference method [34, 35].

The mean age of our study population is 77 years and our cohort might represent a selection of participants that are healthier than the general population. Participants had to recall their dietary habits many decades back in time which could result in a misclassification of intake. Although, a previous study showed that food-related memory from childhood can be as accurate as from current diet, particularly for foods items eaten daily or rarely [52]. However, participants did not know their MGUS status at the time of questioning, and we therefore assume that the misclassification is non-differential. We did not have data on family history of hematologic cancers or information on total energy intake and were therefore unable to adjust for these factors. We do not know the true time of MGUS onset and therefore a misclassification of follow-up times could be present in our progression analysis, however, the prevalence of MGUS increases with age and by adjusting all our models for age we try to reduce the effect of this misclassification. Another limitation is lack of correlation to the reference method for midlife and late life intake of few food groups when the questionnaire was validated, possibly due to the inability of the food record used as reference method to adequately reflect individual intake of food items that are consumed less than four times a week [34, 35]. Validating early diet does pose great challenges and it is expected that such studies are unable to follow ideal procedures. Interpretation of our results are therefore limited by the results of the validation studies. Adolescent diet has not and cannot be validated, however, the data show similar frequency of intake according to residence in rural and coastal fishing areas, as documented in an Icelandic household study from 1939 [28, 53]. We cannot truly distinguish what could be smoldering multiple myeloma as we do not have bone marrow samples. Lastly, we cannot rule out the option that our findings are due to chance. Some of our results from the adolescent and midlife periods are limited by few number of MGUS or LC-MGUS cases in some categories, our progression analyses are additionally limited by few number of cases. Little is known about the relationship between diet and MGUS/LC-MGUS and progression to MM and this study can therefore be considered a hypothesis-generating study. Adjusting for multiple comparison is thought of as an insurance policy against mistakenly rejecting a null hypothesis, given that the null hypothesis is correct [54]. Due to the number of tests performed in the study the chances of rejecting a null hypothesis and obtaining positive results is high. However, the nature of our study is to seek potential risk factors for MGUS/LC-MGUS and progression to MM and we did not adjust for multiple testing in order not to increase the risk of missing out on possible risk factors.

To conclude, in this population-based screening study we found that high intake of fruit during the adolescent period and whole wheat bread during the midlife period may reduce the risk of MGUS later in life and that high fruit intake in late life may reduce the risk of progressing from MGUS/LC-MGUS to MM. Our findings suggest that food intake might alter the risk of developing MGUS and progressing to MM. Future studies should focus on clarifying the possible role of dietary habits in the pathogenesis of MGUS and MM.

## Supporting information

S1 Table Longitudinal effect of adolescent and midlife consumption of selected types of food on MGUS. (DOCX)

S2 Table Longitudinal effect of adolescent and midlife consumption of selected types of food on risk of MGUS using higher cutoff values for involved light chains in LC-MGUS cases. (DOCX)

S1 File Survey questionnaire. (PDF)

## References

[1] Landgren O, Kyle RA, Pfeiffer RM, Katzmann JA, Caporaso NE, Hayes RB, et al Monoclonal gammopathy of undetermined significance (MGUS) consistently precedes multiple myeloma: a prospective study. Blood. 2009 5 28;113(22):5412–7. Pubmed Central PMCID: 2689042. Epub 2009/01/31. 10.1182/blood-2008-12-194241 19179464 PMC2689042

[2] Weiss BM, Abadie J, Verma P, Howard RS, Kuehl WM. A monoclonal gammopathy precedes multiple myeloma in most patients. Blood. 2009 5 28;113(22):5418–22. 10.1182/blood-2008-12-195008 . Pubmed Central PMCID: 2689043. Epub 2009/02/24. 19234139 PMC2689043

[3] Swerdlov S, Campo E, Harris N, Jaffe E, Pileri S, Stein H, et al WHO Classification of Tumours of Heamatopoietic and Lymphoid Tissue Lyon: IARC; 2008.

[4] Dispenzieri A, Katzmann JA, Kyle RA, Larson DR, Melton LJ 3rd, Colby CL, et al Prevalence and risk of progression of light-chain monoclonal gammopathy of undetermined significance: a retrospective population-based cohort study. Lancet. 2010 5 15;375(9727):1721–8. 10.1016/S0140-6736(10)60482-5 . Pubmed Central PMCID: 2904571. Epub 2010/05/18. 20472173 PMC2904571

[5] Eisele L, Durig J, Huttmann A, Duhrsen U, Assert R, Bokhof B, et al Prevalence and progression of monoclonal gammopathy of undetermined significance and light-chain MGUS in Germany. Annals of hematology. 2012 2;91(2):243–8. 10.1007/s00277-011-1293-1 . Epub 2011/07/27. 21789623

[6] Kyle RA, Therneau TM, Rajkumar SV, Larson DR, Plevak MF, Offord JR, et al Prevalence of monoclonal gammopathy of undetermined significance. The New England journal of medicine. 2006 3 30;354(13):1362–9. 10.1056/NEJMoa054494 . Epub 2006/03/31. 16571879

[7] Turesson I, Kovalchik SA, Pfeiffer RM, Kristinsson SY, Goldin LR, Drayson MT, et al Monoclonal gammopathy of undetermined significance and risk of lymphoid and myeloid malignancies: 728 cases followed up to 30 years in Sweden. Blood. 2014 1 16;123(3):338–45. 10.1182/blood-2013-05-505487 . Pubmed Central PMCID: PMC3894492. Epub 2013/11/14. 24222331 PMC3894492

[8] Kyle RA, Therneau TM, Rajkumar SV, Offord JR, Larson DR, Plevak MF, et al A long-term study of prognosis in monoclonal gammopathy of undetermined significance. The New England journal of medicine. 2002 2 21;346(8):564–9. 10.1056/NEJMoa01133202 . Epub 2002/02/22. 11856795

[9] Thordardottir M, Lindqvist EK, Lund SH, Costello R, Burton D, Korde N, et al Obesity and risk of monoclonal gammopathy of undetermined significance and progression to multiple myeloma: a population-based study. Blood Advances. 2017;1(24):2186–92. 10.1182/bloodadvances.2017007609 29296866 PMC5737120

[10] Landgren O, Katzmann JA, Hsing AW, Pfeiffer RM, Kyle RA, Yeboah ED, et al Prevalence of monoclonal gammopathy of undetermined significance among men in Ghana. Mayo Clinic proceedings. 2007 12;82(12):1468–73. 10.1016/S0025-6196(11)61089-6 . Epub 2007/12/07. 18053453

[11] Landgren O, Gridley G, Turesson I, Caporaso NE, Goldin LR, Baris D, et al Risk of monoclonal gammopathy of undetermined significance (MGUS) and subsequent multiple myeloma among African American and white veterans in the United States. Blood. 2006 2 1;107(3):904–6. 10.1182/blood-2005-08-3449 . Pubmed Central PMCID: 1895893. Epub 2005/10/08. 16210333 PMC1895893

[12] Landgren O, Kristinsson SY, Goldin LR, Caporaso NE, Blimark C, Mellqvist UH, et al Risk of plasma cell and lymphoproliferative disorders among 14621 first-degree relatives of 4458 patients with monoclonal gammopathy of undetermined significance in Sweden. Blood. 2009 7 23;114(4):791–5. 10.1182/blood-2008-12-191676 . Pubmed Central PMCID: PMC2716021. Epub 2009/02/03. 19182202 PMC2716021

[13] Brown LM, Gridley G, Check D, Landgren O. Risk of multiple myeloma and monoclonal gammopathy of undetermined significance among white and black male United States veterans with prior autoimmune, infectious, inflammatory, and allergic disorders. Blood. 2008 4 1;111(7):3388–94. 10.1182/blood-2007-10-121285 . Pubmed Central PMCID: 2275008. Epub 2008/02/02. 18239085 PMC2275008

[14] Lindqvist EK, Goldin LR, Landgren O, Blimark C, Mellqvist UH, Turesson I, et al Personal and family history of immune-related conditions increase the risk of plasma cell disorders: a population-based study. Blood. 2011 12 8;118(24):6284–91. 10.1182/blood-2011-04-347559 . Pubmed Central PMCID: PMC3236117. Epub 2011/10/15. 21998210 PMC3236117

[15] Landgren O, Shim YK, Michalek J, Costello R, Burton D, Ketchum N, et al Agent Orange Exposure and Monoclonal Gammopathy of Undetermined Significance: An Operation Ranch Hand Veteran Cohort Study. JAMA oncology. 2015 11;1(8):1061–8. 10.1001/jamaoncol.2015.2938 . Epub 2015/09/04. 26335650 PMC5701511

[16] Baris D, Brown LM, Silverman DT, Hayes R, Hoover RN, Swanson GM, et al Socioeconomic status and multiple myeloma among US blacks and whites. American journal of public health. 2000 8;90(8):1277–81. PubMed . Pubmed Central PMCID: 1446323. Epub 2000/08/11. 10937009 10.2105/ajph.90.8.1277 PMC1446323

[17] Wallin A, Larsson SC. Body mass index and risk of multiple myeloma: a meta-analysis of prospective studies. European journal of cancer (Oxford, England: 1990). 2011 7;47(11):1606–15. 10.1016/j.ejca.2011.01.020 . Epub 2011/03/01. 21354783

[18] Hofmann JN, Moore SC, Lim U, Park Y, Baris D, Hollenbeck AR, et al Body mass index and physical activity at different ages and risk of multiple myeloma in the NIH-AARP diet and health study. American journal of epidemiology. 2013 4 15;177(8):776–86. 10.1093/aje/kws295 . Pubmed Central PMCID: PMC3668425. Epub 2013/04/02. 23543160 PMC3668425

[19] Lauby-Secretan B, Scoccianti C, Loomis D, Grosse Y, Bianchini F, Straif K. Body Fatness and Cancer—Viewpoint of the IARC Working Group. The New England journal of medicine. 2016 8 25;375(8):794–8. 10.1056/NEJMsr1606602 . Epub 2016/08/25. 27557308 PMC6754861

[20] Tavani A, La Vecchia C, Gallus S, Lagiou P, Trichopoulos D, Levi F, et al Red meat intake and cancer risk: a study in Italy. Int J Cancer. 2000 5 1;86(3):425–8. PubMed . Epub 2000/04/13. 10760833 10.1002/(sici)1097-0215(20000501)86:3<425::aid-ijc19>3.0.co;2-s

[21] Brown LM, Gridley G, Pottern LM, Baris D, Swanso CA, Silverman DT, et al Diet and nutrition as risk factors for multiple myeloma among blacks and whites in the United States. Cancer causes & control: CCC. 2001 2;12(2):117–25. PubMed . Epub 2001/03/15. 11246840 10.1023/a:1008937901586

[22] Vlajinac HD, Pekmezovic TD, Adanja BJ, Marinkovic JM, Kanazir MS, Suvajdzic ND, et al Case-control study of multiple myeloma with special reference to diet as risk factor. Neoplasma. 2003;50(1):79–83. PubMed . Epub 2003/04/11. 12687283

[23] Hosgood HD 3rd, Baris D, Zahm SH, Zheng T, Cross AJ. Diet and risk of multiple myeloma in Connecticut women. Cancer causes & control: CCC. 2007 12;18(10):1065–76. 10.1007/s10552-007-9047-z . Epub 2007/08/19. 17694422

[24] Tavani A, Pregnolato A, Negri E, Franceschi S, Serraino D, Carbone A, et al Diet and risk of lymphoid neoplasms and soft tissue sarcomas. Nutrition and cancer. 1997;27(3):256–60. 10.1080/01635589709514535 . Epub 1997/01/01. 9101555

[25] Fernandez E, Chatenoud L, La Vecchia C, Negri E, Franceschi S. Fish consumption and cancer risk. The American journal of clinical nutrition. 1999 7;70(1):85–90. 10.1093/ajcn/70.1.85 . Epub 1999/07/07. 10393143

[26] Fritschi L, Ambrosini GL, Kliewer EV, Johnson KC. Dietary fish intake and risk of leukaemia, multiple myeloma, and non-Hodgkin lymphoma. Cancer epidemiology, biomarkers & prevention: a publication of the American Association for Cancer Research, cosponsored by the American Society of Preventive Oncology. 2004 4;13(4):532–7. PubMed . Epub 2004/04/07. 15066916

[27] Landgren O, Graubard BI, Kumar S, Kyle RA, Katzmann JA, Murata K, et al Prevalence of myeloma precursor state monoclonal gammopathy of undetermined significance in 12372 individuals 10–49 years old: a population-based study from the National Health and Nutrition Examination Survey. Blood cancer journal. 2017 10 20;7(10):e618 10.1038/bcj.2017.97 . Pubmed Central PMCID: PMC5678222. Epub 2017/10/21. 29053158 PMC5678222

[28] Sigurjonsson J. Survey on Diet and Health in Iceland (1939–1940). Reykjavik: 1943.

[29] Steingrimsdottir L, Thorgeirsdottir H, Olafsdottir AS. The Diet of Icelanders. Dietary Survey of The Icelandic Nutrition Council 2002 Main findings. Reykjavik, Iceland: The Directorate of Health, 2003.

[30] Jonsdottir LS, Sigfusson N, Gudnason V, Sigvaldason H, Thorgeirsson G. Do lipids, blood pressure, diabetes, and smoking confer equal risk of myocardial infarction in women as in men? The Reykjavik Study. Journal of cardiovascular risk. 2002 4;9(2):67–76. PubMed . Epub 2002/05/15. 12006913

[31] Sigurdsson E, Thorgeirsson G, Sigvaldason H, Sigfusson N. Prevalence of coronary heart disease in Icelandic men 1968–1986. The Reykjavik Study. European heart journal. 1993 5;14(5):584–91. PubMed . Epub 1993/05/01. 8508850 10.1093/eurheartj/14.5.584

[32] Harris TB, Launer LJ, Eiriksdottir G, Kjartansson O, Jonsson PV, Sigurdsson G, et al Age, Gene/Environment Susceptibility-Reykjavik Study: multidisciplinary applied phenomics. American journal of epidemiology. 2007 5 1;165(9):1076–87. 10.1093/aje/kwk115 . Pubmed Central PMCID: 2723948. Epub 2007/03/14. 17351290 PMC2723948

[33] Torfadottir JE, Valdimarsdottir UA, Mucci LA, Kasperzyk JL, Fall K, Tryggvadottir L, et al Consumption of fish products across the lifespan and prostate cancer risk. PloS one. 2013;8(4):e59799 10.1371/journal.pone.0059799 . Pubmed Central PMCID: PMC3629172. Epub 2013/04/25. 23613715 PMC3629172

[34] Eysteinsdottir T, Gunnarsdottir I, Thorsdottir I, Harris T, Launer LJ, Gudnason V, et al Validity of retrospective diet history: assessing recall of midlife diet using food frequency questionnaire in later life. J Nutr Health Aging. 2011 12;15(10):809–14. PubMed . Epub 2011/12/14. 22159766 10.1007/s12603-011-0067-8 PMC5314731

[35] Eysteinsdottir T, Thorsdottir I, Gunnarsdottir I, Steingrimsdottir L. Assessing validity of a short food frequency questionnaire on present dietary intake of elderly Icelanders. Nutr J. 2012;11:12 10.1186/1475-2891-11-12 . Pubmed Central PMCID: PMC3349496. Epub 2012/03/15. 22413931 PMC3349496

[36] Thorsteinsdottir S, Lund SH, Lindqvist EK, Thordardottir M, Sigurdsson G, Costello R, et al Bone disease in monoclonal gammopathy of undetermined significance: results from a screened population-based study. Blood Advances. 2017;1(27):2790–8. 10.1182/bloodadvances.2017010454 29296931 PMC5745135

[37] Rajkumar SV, Kyle RA, Therneau TM, Melton LJ 3rd, Bradwell AR, Clark RJ, et al Serum free light chain ratio is an independent risk factor for progression in monoclonal gammopathy of undetermined significance. Blood. 2005 8 1;106(3):812–7. 10.1182/blood-2005-03-1038 . 15855274 PMC1895159

[38] Abadie JM, Bankson DD. Assessment of serum free light chain assays for plasma cell disorder screening in a Veterans Affairs population. Annals of clinical and laboratory science. 2006 Spring;36(2):157–62. PubMed . Epub 2006/05/10. 16682511

[39] Criteria for the classification of monoclonal gammopathies, multiple myeloma and related disorders: a report of the International Myeloma Working Group. British journal of haematology. 2003 6;121(5):749–57. PubMed . Epub 2003/06/05. 12780789

[40] Katzmann JA, Clark RJ, Abraham RS, Bryant S, Lymp JF, Bradwell AR, et al Serum reference intervals and diagnostic ranges for free kappa and free lambda immunoglobulin light chains: relative sensitivity for detection of monoclonal light chains. Clinical chemistry. 2002 9;48(9):1437–44. PubMed . Epub 2002/08/27. 12194920

[41] Sigurdardottir LG, Jonasson JG, Stefansdottir S, Jonsdottir A, Olafsdottir GH, Olafsdottir EJ, et al Data quality at the Icelandic Cancer Registry: comparability, validity, timeliness and completeness. Acta oncologica (Stockholm, Sweden). 2012 9;51(7):880–9. 10.3109/0284186X.2012.698751 . Epub 2012/09/15. 22974093

[42] R Core Team. R: A Language and Environment for Statistical Computing Vienna, Austria: R Foundation for Statistical Computing; 2017.

[43] Bradbury KE, Appleby PN, Key TJ. Fruit, vegetable, and fiber intake in relation to cancer risk: findings from the European Prospective Investigation into Cancer and Nutrition (EPIC). The American journal of clinical nutrition. 2014 7;100 Suppl 1:394S–8S. 10.3945/ajcn.113.071357 . Epub 2014/06/13. 24920034

[44] Turati F, Rossi M, Pelucchi C, Levi F, La Vecchia C. Fruit and vegetables and cancer risk: a review of southern European studies. The British journal of nutrition. 2015 4;113 Suppl 2:S102–10. 10.1017/S0007114515000148 . Epub 2015/07/08. 26148912

[45] Lutsenko EA, Carcamo JM, Golde DW. Vitamin C prevents DNA mutation induced by oxidative stress. The Journal of biological chemistry. 2002 5 10;277(19):16895–9. 10.1074/jbc.M201151200 . Epub 2002/03/09. 11884413

[46] Rose DP, Connolly JM. Omega-3 fatty acids as cancer chemopreventive agents. Pharmacology & therapeutics. 1999 9;83(3):217–44. PubMed . Epub 1999/11/27. 10576293 10.1016/s0163-7258(99)00026-1

[47] Darmon N, Drewnowski A. Does social class predict diet quality? The American journal of clinical nutrition. 2008 5;87(5):1107–17. 10.1093/ajcn/87.5.1107 . Epub 2008/05/13. 18469226

[48] Harris TB, Song X, Reinders I, Lang TF, Garcia ME, Siggeirsdottir K, et al Plasma phospholipid fatty acids and fish-oil consumption in relation to osteoporotic fracture risk in older adults: the Age, Gene/Environment Susceptibility Study. The American journal of clinical nutrition. 2015 5;101(5):947–55. 10.3945/ajcn.114.087502 . Pubmed Central PMCID: PMC4409686. Epub 2015/03/20. 25787995 PMC4409686

[49] Chatenoud L, Tavani A, La Vecchia C, Jacobs DR Jr., Negri E, Levi F, et al Whole grain food intake and cancer risk. International journal of cancer Journal international du cancer. 1998 7 3;77(1):24–8. PubMed . Epub 1998/06/25. 9639389 10.1002/(sici)1097-0215(19980703)77:1<24::aid-ijc5>3.0.co;2-1

[50] Mirmiran P, Bahadoran Z, Azizi F. Functional foods-based diet as a novel dietary approach for management of type 2 diabetes and its complications: A review. World journal of diabetes. 2014 6 15;5(3):267–81. 10.4239/wjd.v5.i3.267 . Pubmed Central PMCID: PMC4058731. Epub 2014/06/18. 24936248 PMC4058731

[51] Ferlin M, Noraz N, Hertogh C, Brochier J, Taylor N, Klein B. Insulin-like growth factor induces the survival and proliferation of myeloma cells through an interleukin-6-independent transduction pathway. British journal of haematology. 2000 11;111(2):626–34. PubMed . Epub 2000/12/21. 11122111 10.1046/j.1365-2141.2000.02364.x

[52] Dwyer JT, Coleman KA. Insights into dietary recall from a longitudinal study: accuracy over four decades. The American journal of clinical nutrition. 1997 4;65(4 Suppl):1153S–8S. 10.1093/ajcn/65.4.1153S . Epub 1997/04/01. 9094913

[53] Torfadottir JE, Steingrimsdottir L, Mucci L, Aspelund T, Kasperzyk JL, Olafsson O, et al Milk intake in early life and risk of advanced prostate cancer. American journal of epidemiology. 2012 1 15;175(2):144–53. 10.1093/aje/kwr289 . Pubmed Central PMCID: PMC3249408. Epub 2011/12/23. 22190107 PMC3249408

[54] Rothman KJ. No adjustments are needed for multiple comparisons. Epidemiology (Cambridge, Mass). 1990 1;1(1):43–6. PubMed . Epub 1990/01/01. 2081237

