# Supplementary material for: Dietary intake is associated with risk of multiple myeloma and its precursor disease
Source: PLoS One. 2018 Nov 1;13(11):e0206047. doi: 10.1371/journal.pone.0206047 (PMC6211667; doi:10.1371/journal.pone.0206047)
Supplement: S1 Table — (DOCX) [file pone.0206047.s001.docx]

**S1 Table. Longitudinal effect of adolescent and midlife consumption of selected types of food on MGUS.**

|  | **MGUS*** | **Age and sex adjusted OR** | **95% CI** |
| --- | --- | --- | --- |
|  | **n (%)** |  |  |
| **Fish** |  |  |  |
| Low_adol_ - Low_mid_ | 32 (6.3) | 1.00 |  |
| Low_adol_ - High_mid_ | 207 (40.6) | 0.99 | 0.96-1.03 |
| High_adol_ - Low_mid_ | 36 (7.1) | 1.03 | 0.98-1.07 |
| High_adol_ - High_mid_ | 235 (40.1) | 0.99 | 0.96-1.03 |
| **Fish oil** |  |  |  |
| Low_adol_ - Low_mid_ | 147 (28.8) | 1.00 |  |
| Low_adol_ - High_mid_ | 99 (19.4) | 1.01 | 0.99-1.03 |
| High_adol_ - Low_mid_ | 47 (9.2) | 1.00 | 0.97-1.03 |
| High_adol_ - High_mid_ | 217 (42.5) | 1.00 | 0.98-1.02 |
| **Salted/smoked fish** |  |  |  |
| Low_adol_ - Low_mid_ | 212 (41.5) | 1.00 |  |
| Low_adol_ - High_mid_ | 30 (5.9) | 0.97 | 0.94-1.01 |
| High_adol_ - Low_mid_ | 136 (26.6) | 0.98 | 0.96-1.00 |
| High_adol_ - High_mid_ | 133 (26.0) | 0.98 | 0.96-1.00 |
| **Meat** |  |  |  |
| Low_adol_ - Low_mid_ | 50 (9.8) | 1.00 |  |
| Low_adol_ - High_mid_ | 144 (28.3) | 1.00 | 0.97-1.04 |
| High_adol_ - Low_mid_ | 148 (29.1) | 1.00 | 0.97-1.03 |
| High_adol_ - High_mid_ | 167 (32.8) | 0.99 | 0.96-1.02 |
| **Salted/smoked meat** |  |  |  |
| Low_adol_ - Low_mid_ | 289 (56.8) | 1.00 |  |
| Low_adol_ - High_mid_ | 52 (10.2) | 0.99 | 0.96-1.02 |
| High_adol_ - Low_mid_ | 79 (15.5) | 0.98 | 0.96-1.00 |
| High_adol_ - High_mid_ | 89 (17.5) | 0.99 | 0.97-1.01 |
| **Milk** |  |  |  |
| Low_adol_ - Low_mid_ | 91 (17.9) | 1.00 |  |
| Low_adol_ - High_mid_ | 35 (6.9) | 1.03 | 0.99-1.07 |
| High_adol_ - Low_mid_ | 98 (19.3) | 0.98 | 0.96-1.01 |
| High_adol_ - High_mid_ | 285 (56.0) | 0.99 | 0.97-1.02 |
| **Fruit** |  |  |  |
| Low_adol_ - Low_mid_ | 339 (66.5) | 1.00 |  |
| Low_adol_ - High_mid_ | 119 (23.3) | 1.00 | 0.99-1.02 |
| High_adol_ - Low_mid_ | 26 (5.1) | 0.98 | 0.95-1.01 |
| High_adol_ - High_mid_ | 26 (5.1) | 0.98 | 0.94-1.01 |
| **Vegetables** |  |  |  |
| Low_adol_ - Low_mid_ | 291 (57.2) | 1.00 |  |
| Low_adol_ - High_mid_ | 102 (20.0) | 1.00 | 0.98-1.02 |
| High_adol_ - Low_mid_ | 52 (10.2) | 0.99 | 0.96-1.01 |
| High_adol_ - High_mid_ | 64 (12.6) | 0.99 | 0.97-1.02 |
| **Rye bread/flatbread** |  |  |  |
| Low_adol_ - Low_mid_ | 237 (46.4) | 1.00 |  |
| Low_adol_ - High_mid_ | 24 (4.7) | 0.98 | 0.95-1.02 |
| High_adol_ - Low_mid_ | 114 (22.3) | 1.00 | 0.98-1.02 |
| High_adol_ - High_mid_ | 136 (26.6) | 0.98 | 0.96-1.00 |
| **Liver sausage** |  |  |  |
| Low_adol_ - Low_mid_ | 124 (24.3) | 1.00 |  |
| Low_adol_ - High_mid_ | 18 (3.5) | 0.97 | 0.93-1.01 |
| High_adol_ - Low_mid_ | 111 (21.7) | 0.97 | 0.94-0.99 |
| High_adol_ - High_mid_ | 258 (50.5) | 0.98 | 0.96-1.00 |
| **Oatmeal** |  |  |  |
| Low_adol_ - Low_mid_ | 176 (34.7) | 1.00 |  |
| Low_adol_ - High_mid_ | 41 (8.1) | 1.00 | 0.97-1.03 |
| High_adol_ - Low_mid_ | 111 (21.9) | 0.99 | 0.97-1.01 |
| High_adol_ - High_mid_ | 179 (35.3) | 1.00 | 0.98-1.02 |
| **Potatoes** |  |  |  |
| Low_adol_ - Low_mid_ | 41 (8.0) | 1.00 |  |
| Low_adol_ - High_mid_ | 19 (3.7) | 0.97 | 0.92-1.02 |
| High_adol_ - Low_mid_ | 47 (9.2) | 0.98 | 0.94-1.02 |
| High_adol_ - High_mid_ | 403 (79.0) | 0.97 | 0.94-1.00 |

*Heavy chain and light chain MGUS cases combined.

Abbreviations: MGUS - Monoclonal gammopathy of undetermined significance.

The low and high categories represent the same frequency of intake as in Tables 2a and 2b.
