# Supplementary material for: Dietary intake is associated with risk of multiple myeloma and its precursor disease
Source: PLoS One. 2018 Nov 1;13(11):e0206047. doi: 10.1371/journal.pone.0206047 (PMC6211667; doi:10.1371/journal.pone.0206047)
Supplement: S2 Table — (DOCX) [file pone.0206047.s002.docx]

**S2 Table. Longitudinal effect of adolescent and midlife consumption of selected types of food on risk of MGUS using higher cutoff values for involved light chains in LC-MGUS cases.**

|  | **MGUS*** | **Age and sex adjusted OR** | **95% CI** |
| --- | --- | --- | --- |
|  | **n (%)** |  |  |
| **Fish** |  |  |  |
| Low_adol_ - Low_mid_ | 21 (6.8) | 1.00 |  |
| Low_adol_ - High_mid_ | 139 (45.0) | 0.94 | 0.59-1.55 |
| High_adol_ - Low_mid_ | 19 (6.1) | 1.00 | 0.52-1.90 |
| High_adol_ - High_mid_ | 130 (42.1) | 0.78 | 0.49-1.29 |
| **Fish oil** |  |  |  |
| Low_adol_ - Low_mid_ | 84 (27.3) | 1.00 |  |
| Low_adol_ - High_mid_ | 56 (18.2) | 1.08 | 0.76-1.53 |
| High_adol_ - Low_mid_ | 31 (10.1) | 1.22 | 0.79-1.86 |
| High_adol_ - High_mid_ | 137 (44.5) | 1.11 | 0.84-1.48 |
| **Salted/smoked fish** |  |  |  |
| Low_adol_ - Low_mid_ | 131 (42.4) | 1.00 |  |
| Low_adol_ - High_mid_ | 15 (4.9) | 0.61 | 0.34-1.02 |
| High_adol_ - Low_mid_ | 82 (26.5) | 0.81 | 0.61-1.08 |
| High_adol_ - High_mid_ | 81 (26.2) | 0.80 | 0.60-1.08 |
| **Meat** |  |  |  |
| Low_adol_ - Low_mid_ | 34 (11.0) | 1.00 |  |
| Low_adol_ - High_mid_ | 85 (27.6) | 0.89 | 0.59-1.37 |
| High_adol_ - Low_mid_ | 94 (30.5) | 0.90 | 0.60-1.36 |
| High_adol_ - High_mid_ | 95 (30.8) | 0.73 | 0.49-1.10 |
| **Salted/smoked meat** |  |  |  |
| Low_adol_ - Low_mid_ | 178 (57.8) | 1.00 |  |
| Low_adol_ - High_mid_ | 32 (10.4) | 0.91 | 0.61-1.33 |
| High_adol_ - Low_mid_ | 50 (16.2) | 0.78 | 0.56-1.08 |
| High_adol_ - High_mid_ | 48 (15.6) | 0.78 | 0.56-1.08 |
| **Milk** |  |  |  |
| Low_adol_ - Low_mid_ | 58 (18.8) | 1.00 |  |
| Low_adol_ - High_mid_ | 19 (6.2) | 1.07 | 0.61-1.81 |
| High_adol_ - Low_mid_ | 60 (19.5) | 0.78 | 0.53-1.13 |
| High_adol_ - High_mid_ | 171 (55.5) | 0.88 | 0.65-1.23 |
| **Fruit** |  |  |  |
| Low_adol_ - Low_mid_ | 198 (64.1) | 1.00 |  |
| Low_adol_ - High_mid_ | 77 (24.9) | 1.18 | 0.89-1.55 |
| High_adol_ - Low_mid_ | 17 (5.5) | 0.88 | 0.51-1.43 |
| High_adol_ - High_mid_ | 17 (5.5) | 0.80 | 0.47-1.30 |
| **Vegetables** |  |  |  |
| Low_adol_ - Low_mid_ | 176 (57.3) | 1.00 |  |
| Low_adol_ - High_mid_ | 56 (18.2) | 0.88 | 0.64-1.20 |
| High_adol_ - Low_mid_ | 35 (11.4) | 0.97 | 0.66-1.40 |
| High_adol_ - High_mid_ | 40 (13.0) | 0.94 | 0.65-1.33 |
| **Rye bread/flatbread** |  |  |  |
| Low_adol_ - Low_mid_ | 144 (46.6) | 1.00 |  |
| Low_adol_ - High_mid_ | 21 (6.8) | 1.24 | 0.75-1.96 |
| High_adol_ - Low_mid_ | 74 (23.9) | 1.03 | 0.76-1.38 |
| High_adol_ - High_mid_ | 70 (22.7) | 0.70 | 0.55-0.95 |
| **Liver sausage** |  |  |  |
| Low_adol_ - Low_mid_ | 76 (24.6) | 1.00 |  |
| Low_adol_ - High_mid_ | 9 (2.9) | 0.58 | 0.26-1.11 |
| High_adol_ - Low_mid_ | 63 (20.4) | 0.61 | 0.44-0.87 |
| High_adol_ - High_mid_ | 161 (52.1) | 0.87 | 0.65-1.16 |
| **Oatmeal** |  |  |  |
| Low_adol_ - Low_mid_ | 103 (33.6) | 1.00 |  |
| Low_adol_ - High_mid_ | 24 (7.8) | 1.03 | 0.64-1.61 |
| High_adol_ - Low_mid_ | 73 (23.8) | 0.97 | 0.71-1.32 |
| High_adol_ - High_mid_ | 107 (34.9) | 1.04 | 0.78-1.39 |
| **Potatoes** |  |  |  |
| Low_adol_ - Low_mid_ | 29 (9.4) | 1.00 |  |
| Low_adol_ - High_mid_ | 15 (4.9) | 0.83 | 0.42-1.57 |
| High_adol_ - Low_mid_ | 26 (8.4) | 0.65 | 0.37-1.13 |
| High_adol_ - High_mid_ | 239 (77.3) | 0.63 | 0.45-0.96 |

*Heavy chain MGUS and light chain MGUS cases combined.

Abbreviations: MGUS - Monoclonal gammopathy of undetermined significance.

The low and high categories represent the same frequency of intake as in Tables 2a and 2b.
