# Supplementary material for: Dietary intake is associated with risk of multiple myeloma and its precursor disease
Source: PLoS One. 2018 Nov 1;13(11):e0206047. doi: 10.1371/journal.pone.0206047 (PMC6211667; doi:10.1371/journal.pone.0206047)
Supplement: S1 File — (PDF) [file pone.0206047.s003.pdf]

16 DATA TO BE REGISTERED IN RECEPTION FOR VISIT 2

The food questionnaire is received in the reception for the second visit and also the second part of questionnaire 1 (see definitions in Reception 1). The lists are scanned into the database. There are the following lists defined and the second one is registered manually into the RIS system:

Questionnaire 2	Table 16.1	Page 16.2
Results of questionnaire registration	Table 16.2	Page 16.37

Table 16.1 Questionnaire 2 (Q2_FOODLIST).

Name of variable	Type	Length	Number of decimal figures	Not Null (Y/N)	Max value	Min value	Other constraints	Units	Description
QUE2PROJ	Varchar2	10	-	Y	-	-	Foreign key into table of allowed values	-	IHA project code
QUE2S_ID	Varchar2	10	-	Y	-	-	Primary key	-	The subject ID that is used throughout the study and printed on the ID card
QUE2DATE	Date	-	-	Y	-	-		-	Date of test
QUE2P_ID	Varchar2	10	-	Y	-	-	-	-	The personal ID given to all AGES personnel
QUE2NHML	Number	1	0	N	7	1	-	-	On average, how often do you eat a hot main meal?Q1<br>1 = Never<br>2 = Less than once a week<br>3 = 1-2 times a week<br>4 = 3-4 times a week<br>5 = 5-6 times a week<br>6 = Daily<br>7 = More than once a day
QUE2NMMM	Number	1	0	N	7	1	-	-	On average, how often do you eat meat or a ground meat dish for a main meal? Q2<br>1 = Never<br>2 = Less than once a week<br>3 = 1-2 times a week<br>4 = 3-4 times a week<br>5 = 5-6 times a week<br>6 = Daily<br>7 = More than once a day

Name of variable	Type	Length	Number of decimal figures	Not Null (Y/N)	Max value	Min value	Other constraints	Units	Description
QUE2NFMM	Number	1	0	N	7	1	-	-	On average, how often do you eat fish or a fish dish for a main meal? Q3<br>1 = Never<br>2 = Less than once a week<br>3 = 1-2 times a week<br>4 = 3-4 times a week<br>5 = 5-6 times a week<br>6 = Daily<br>7 = More than once a day
QUE2NFBR	Number	1	0	N	7	1	-	-	On average, how often do you eat fish on a bread or in a salad such as or sardines, trout or salmon? Q4<br>1 = Never<br>2 = Less than once a week<br>3 = 1-2 times a week<br>4 = 3-4 times a week<br>5 = 5-6 times a week<br>6 = Daily<br>7 = More than once a day
QUE2NPOT	Number	1	0	N	7	1	-	-	On average, how often do you eat potatoes? Q5<br>1 = Never<br>2 = Less than once a week<br>3 = 1-2 times a week<br>4 = 3-4 times a week<br>5 = 5-6 times a week<br>6 = Daily<br>7 = More than once a day

Name of variable	Type	Length	Number of decimal figures	Not Null (Y/N)	Max value	Min value	Other constraints	Units	Description
QUE2NFFR	Number	1	0	N	7	1	-	-	On average, how often do you eat fresh fruit? Q6<br>1 = Never<br>2 = Less than once a week<br>3 = 1-2 times a week<br>4 = 3-4 times a week<br>5 = 5-6 times a week<br>6 = Daily<br>7 = More than once a day
QUE2NBLS	Number	1	0	N	7	1	-	-	On average, how often do you eat blood sausage or liver sausage? Q7<br>1 = Never<br>2 = Less than once a week<br>3 = 1-2 times a week<br>4 = 3-4 times a week<br>5 = 5-6 times a week<br>6 = Daily<br>7 = More than once a day
QUE2NRYB	Number	1	0	N	7	1	-	-	On average, how often do you eat rye bread or flatbread (made from rye)? Q8<br>1 = Never<br>2 = Less than once a week<br>3 = 1-2 times a week<br>4 = 3-4 times a week<br>5 = 5-6 times a week<br>6 = Daily<br>7 = More than once a day

Name of variable	Type	Length	Number of decimal figures	Not Null (Y/N)	Max value	Min value	Other constraints	Units	Description
QUE2NWHB	Number	1	0	N	7	1	-	-	On average, how often do you eat whole-wheat bread or another kind of coarse-grain bread? Q9<br>1 = Never<br>2 = Less than once a week<br>3 = 1-2 times a week<br>4 = 3-4 times a week<br>5 = 5-6 times a week<br>6 = Daily<br>7 = More than once a day
QUE2NOAT	Number	1	0	N	7	1	-	-	On average, how often do you eat oatmeal or muesli?Q10<br>1 = Never<br>2 = Less than once a week<br>3 = 1-2 times a week<br>4 = 3-4 times a week<br>5 = 5-6 times a week<br>6 = Daily<br>7 = More than once a day
QUE2NBVG	Number	1	0	N	7	1	-	-	On average, how often do you eat boiled or fried vegetables? Q11<br>1 = Never<br>2 = Less than once a week<br>3 = 1-2 times a week<br>4 = 3-4 times a week<br>5 = 5-6 times a week<br>6 = Daily<br>7 = More than once a day

Name of variable	Type	Length	Number of decimal figures	Not Null (Y/N)	Max value	Min value	Other constraints	Units	Description
QUE2NRVG	Number	1	0	N	7	1	-	-	On average, how often do you eat a vegetable salad or other kinds of raw vegetables? Q12<br>1 = Never<br>2 = Less than once a week<br>3 = 1-2 times a week<br>4 = 3-4 times a week<br>5 = 5-6 times a week<br>6 = Daily<br>7 = More than once a day
QUE2NCAK	Number	1	0	N	7	1	-	-	On average, how often do you eat cookies, cakes or pastry? Q12<br>1 = Never<br>2 = Less than once a week<br>3 = 1-2 times a week<br>4 = 3-4 times a week<br>5 = 5-6 times a week<br>6 = Daily<br>7 = More than once a day
QUE2NCND	Number	1	0	N	7	1	-	-	On average, how often do you eat candy? Q13<br>1 = Never<br>2 = Less than once a week<br>3 = 1-2 times a week<br>4 = 3-4 times a week<br>5 = 5-6 times a week<br>6 = Daily<br>7 = More than once a day

Name of variable	Type	Length	Number of decimal figures	Not Null (Y/N)	Max value	Min value	Other constraints	Units	Description
QUE2NMPR	Number	1	0	N	7	1	-	-	On average, how often do you eat cultured milk products, skyr (an Icelandic milk-curd product) or milk gruel or porridge? Q15<br>1 = Never<br>2 = Less than once a week<br>3 = 1-2 times a week<br>4 = 3-4 times a week<br>5 = 5-6 times a week<br>6 = Daily<br>7 = More than once a day
QUE2NCMP	Number	1	0	N	4	1	-	-	What kind of cultured milk products do you use most? Q16<br>1 = Low-fat products, such as light AB-milk, light yoghurt or light cultured milk<br>2 = Regular AB-milk, cultured milk or yoghurt<br>3 = High-fat cultured milk products<br>4 = Seldom or never use cultured milk products
QUE2NMLK	Number	1	0	N	7	1	-	-	On average, how often do you drink milk (whole milk, low-fat milk, skim milk, 1%-low-fat milk)? Q17<br>1 = Never<br>2 = Less than once a week<br>3 = 1-2 times a week<br>4 = 3-4 times a week<br>5 = 5-6 times a week<br>6 = Daily<br>7 = More than once a day

Name of variable	Type	Length	Number of decimal figures	Not Null (Y/N)	Max value	Min value	Other constraints	Units	Description
QUE2NTOM	Number	1	0	N	5	1	-	-	What kind of milk do you use most? Q18<br>1 = Low-fat milk<br>2 = Whole-milk<br>3 = Skim milk<br>4 = Vitamin improved light-milk<br>5 = Never use milk
QUE2NPFJ	Number	1	0	N	7	1	-	-	On average, how often do you drink pure fruit juice? Q19<br>1 = Never<br>2 = Less than once a week<br>3 = 1-2 times a week<br>4 = 3-4 times a week<br>5 = 5-6 times a week<br>6 = Daily<br>7 = More than once a day
QUE2NSWD	Number	1	0	N	7	1	-	-	On average, how often do you drink sweetened juice or carbonated beverages? Q20<br>1 = Never<br>2 = Less than once a week<br>3 = 1-2 times a week<br>4 = 3-4 times a week<br>5 = 5-6 times a week<br>6 = Daily<br>7 = More than once a day
QUE2NCLO	Number	1	0	N	6	1	-	-	On average, how often do you take cod or Saithe liver oil or liver oil pills (not halibut liver oil pills)? Q21<br>1 = Never<br>2 = Less than once a week<br>3 = 1-2 times a week<br>4 = 3-4 times a week<br>5 = 5-6 times a week<br>6 = Daily

Name of variable	Type	Length	Number of decimal figures	Not Null (Y/N)	Max value	Min value	Other constraints	Units	Description
QUE2NCOF	Number	1	0	N	6	1	-	-	On average, how many cups of coffee do you drink per day? Q22<br>1 = Never<br>2 = Less than one cup a day<br>3 = 1-2 cups a day<br>4 = 3-4 cups a day<br>5 = 5-6 cups a day<br>6 = 7 or more cups a day
QUE2NTEA	Number	1	0	N	6	1	-	-	On average, how many cups of tea do you drink per day? Q23<br>1 = Never<br>2 = Less than one cup a day<br>3 = 1-2 cups a day<br>4 = 3-4 cups a day<br>5 = 5-6 cups a day<br>6 = 7 or more cups a day
QUE2NCSU	Number	1	0	N	6	1	-	-	How many teaspoons of sugar do you use in coffee or tea per day? Q24<br>1 = Never<br>2 = Less than one spoon a day<br>3 = 1-2 spoons a day<br>4 = 3-4 spoons a day<br>5 = 5-6 spoons a day<br>6 = 7 or more spoons a day
QUE2NSPR	Number	1	0	N	5	1	-	-	What kind of spread do you use most on bread or with food? Q25<br>1 = Reduced-fat butter-oil blend<br>2 = Reduced fat margarines<br>3 = Butter, butter-oil blend<br>4 = Other spreads<br>5 = Don’t use spreads

Name of variable	Type	Length	Number of decimal figures	Not Null (Y/N)	Max value	Min value	Other constraints	Units	Description
QUE2NSMT	Number	1	0	N	6	1	-	-	On average, how often do you eat corned meat, corned meat sausage or another kind of salted/smoked meat? Q26<br>1 = Never<br>2 = Less than once a month<br>3 = 1-3 times a month<br>4 = 1-2 times a week<br>5 = 3-6 times a week<br>6 = Daily or more often
QUE2NSFS	Number	1	0	N	6	1	-	-	On average, how often do you eat salted or smoked fish? Q27<br>1 = Never<br>2 = Less than once a month<br>3 = 1-3 times a month<br>4 = 1-2 times a week<br>5 = 3-6 times a week<br>6 = Daily or more often
QUE2NSLT	Number	1	0	N	3	1	-	-	How much salt to you use on food? Mark the statement that is characteristic of you. Q28<br>1 = I usually sprinkle salt on my food before I start eating<br>2 = I sometimes salt my food on the plate<br>3 = I seldom or never salt my food on the plate
QUE2NSRS	Number	1	0	N	4	1	-	-	Do you generally find the food you get in a cafeteria or restaurant … Q29<br>1 = Not sufficiently salted<br>2 = Adequately salted<br>3 = Too salty<br>4 = Goes seldom to cafeteria or restaurants

Name of variable	Type	Length	Number of decimal figures	Not Null (Y/N)	Max value	Min value	Other constraints	Units	Description
QUE2NBTR	Number	1	0	N	4	1	-	-	How do you generally butter your bread? (Pictures) Q30<br>1 = Little butter<br>2 = Medium butter<br>3 = Much butter<br>4 = Usually doesn’t butter bread
Middle age, 40-50									
QUE2MMMM	Number	1	0	N	7	1	-	-	On average, how often did you eat meat or a ground meat dish for a main meal when you were middle aged? Q31<br>1 = Never<br>2 = Less than once a week<br>3 = 1-2 times a week<br>4 = 3-4 times a week<br>5 = 5-6 times a week<br>6 = Daily<br>7 = More than once a day
QUE2MFMM	Number	1	0	N	7	1	-	-	On average, how often did you eat fish or a fish dish for a main meal when you were middle aged? Q32<br>1 = Never<br>2 = Less than once a week<br>3 = 1-2 times a week<br>4 = 3-4 times a week<br>5 = 5-6 times a week<br>6 = Daily<br>7 = More than once a day

Name of variable	Type	Length	Number of decimal figures	Not Null (Y/N)	Max value	Min value	Other constraints	Units	Description
QUE2MFBR	Number	1	0	N	7	1	-	-	On average, how often do you eat fish on a bread or in a salad such as or sardines, trout or salmon when you were middle aged? Q33<br>1 = Never<br>2 = Less than once a week<br>3 = 1-2 times a week<br>4 = 3-4 times a week<br>5 = 5-6 times a week<br>6 = Daily<br>7 = More than once a day
QUE2MPOT	Number	1	0	N	7	1	-	-	On average, how often did you eat potatoes when you were middle aged? Q34<br>1 = Never<br>2 = Less than once a week<br>3 = 1-2 times a week<br>4 = 3-4 times a week<br>5 = 5-6 times a week<br>6 = Daily<br>7 = More than once a day
QUE2MFFR	Number	1	0	N	7	1	-	-	On average, how often did you eat fresh fruit when you were middle aged? Q35<br>1 = Never<br>2 = Less than once a week<br>3 = 1-2 times a week<br>4 = 3-4 times a week<br>5 = 5-6 times a week<br>6 = Daily<br>7 = More than once a day

Name of variable	Type	Length	Number of decimal figures	Not Null (Y/N)	Max value	Min value	Other constraints	Units	Description
QUE2MBLS	Number	1	0	N	7	1	-	-	On average, how often did you eat blood sausage or liver sausage when you were middle aged? Q36<br>1 = Never<br>2 = Less than once a week<br>3 = 1-2 times a week<br>4 = 3-4 times a week<br>5 = 5-6 times a week<br>6 = Daily<br>7 = More than once a day
QUE2MRYB	Number	1	0	N	7	1	-	-	On average, how often did you eat rye bread or flatbread when you were middle aged? Q37<br>1 = Never<br>2 = Less than once a week<br>3 = 1-2 times a week<br>4 = 3-4 times a week<br>5 = 5-6 times a week<br>6 = Daily<br>7 = More than once a day
QUE2MWHB	Number	1	0	N	7	1	-	-	On average, how often did you eat whole-wheat bread or another kind of coarse bread when you were middle aged? Q38<br>1 = Never<br>2 = Less than once a week<br>3 = 1-2 times a week<br>4 = 3-4 times a week<br>5 = 5-6 times a week<br>6 = Daily<br>7 = More than once a day

Name of variable	Type	Length	Number of decimal figures	Not Null (Y/N)	Max value	Min value	Other constraints	Units	Description
QUE2MOAT	Number	1	0	N	7	1	-	-	On average, how often did you eat oatmeal or muesli when you were middle aged? Q39<br>1 = Never<br>2 = Less than once a week<br>3 = 1-2 times a week<br>4 = 3-4 times a week<br>5 = 5-6 times a week<br>6 = Daily<br>7 = More than once a day
QUE2MVGT	Number	1	0	N	7	1	-	-	On average, how often did you eat vegetables when you were middle aged? Q40<br>1 = Never<br>2 = Less than once a week<br>3 = 1-2 times a week<br>4 = 3-4 times a week<br>5 = 5-6 times a week<br>6 = Daily<br>7 = More than once a day
QUE2MMPR	Number	1	0	N	7	1	-	-	On average, how often did you drink milk or eat milk products when you were middle aged? Q41<br>1 = Never<br>2 = Less than once a week<br>3 = 1-2 times a week<br>4 = 3-4 times a week<br>5 = 5-6 times a week<br>6 = Daily<br>7 = More than once a day

Name of variable	Type	Length	Number of decimal figures	Not Null (Y/N)	Max value	Min value	Other constraints	Units	Description
QUE2MTOM	Number	1	0	N	5	1	-	-	What kind of milk did you use most when you were middle aged? Q42<br>1 = Low fat milk<br>2 = Whole-milk<br>3 = Skim milk<br>4 = Whey<br>5 = Never used milk
QUE2MCLO	Number	1	0	N	7	1	-	-	On average, how often did you take cod or Saithe liver oil or liver oil pills (not halibut liver oil pills) when you were middle aged? Q43<br>1 = Never<br>2 = Less than once a week<br>3 = 1-2 times a week<br>4 = 3-4 times a week<br>5 = 5-6 times a week<br>6 = Daily
QUE2MSMT	Number	1	0	N	6	1	-	-	On average, how often did you eat corned meat, corned meat sausage or another kind of salted/smoked meat when you were middle aged? Q44<br>1 = Never<br>2 = Less than once a month<br>3 = 1-3 times a month<br>4 = 1-2 times a week<br>5 = 3-6 times a week<br>6 = Daily or more often

Name of variable	Type	Length	Number of decimal figures	Not Null (Y/N)	Max value	Min value	Other constraints	Units	Description
QUE2MSFS	Number	1	0	N	6	1	-	-	On average, how often did you eat salt fish or smoked fish when you were middle aged? Q45<br>1 = Never<br>2 = Less than once a month<br>3 = 1-3 times a month<br>4 = 1-2 times a week<br>5 = 3-6 times a week<br>6 = Daily or more often
QUE2MSPR	Number	1	0	N	4	1	-	-	What kind of spread did you use most on bread or with food when you were middle aged? Q46<br>1 = Table margarine, for example Solbloma<br>2 = Regular margarine<br>3 = Butter<br>4 = Didn’t use spread
QUE2MBTR	Number	1	0	N	4	1	-	-	How did you generally butter your bread when you were middle aged? (Pictures) Q47<br>1 = Little butter<br>2 = Medium butter<br>3 = Much butter<br>4 = Usually didn’t butter bread
Teenager, 14-19									
QUE2TMMM	Number	1	0	N	7	1	-	-	On average, how often did you eat meat or a ground meat dish for a main meal when you were a teenager? Q48<br>1 = Never<br>2 = Less than once a week<br>3 = 1-2 times a week<br>4 = 3-4 times a week<br>5 = 5-6 times a week<br>6 = Daily<br>7 = More than once a day

Name of variable	Type	Length	Number of decimal figures	Not Null (Y/N)	Max value	Min value	Other constraints	Units	Description
QUE2TFMM	Number	1	0	N	7	1	-	-	On average, how often did you eat fish or a fish dish for a main meal when you were a teenager? Q49<br>1 = Never<br>2 = Less than once a week<br>3 = 1-2 times a week<br>4 = 3-4 times a week<br>5 = 5-6 times a week<br>6 = Daily<br>7 = More than once a day
QUE2TFBR	Number	1	0	N	7	1	-	-	On average, how often do you eat fish on a bread or in a salad such as or sardines, trout or salmon when you were a teenager? Q50<br>1 = Never<br>2 = Less than once a week<br>3 = 1-2 times a week<br>4 = 3-4 times a week<br>5 = 5-6 times a week<br>6 = Daily<br>7 = More than once a day
QUE2TPOT	Number	1	0	N	7	1	-	-	On average, how often did you eat potatoes when you were a teenager? Q51<br>1 = Never<br>2 = Less than once a week<br>3 = 1-2 times a week<br>4 = 3-4 times a week<br>5 = 5-6 times a week<br>6 = Daily<br>7 = More than once a day

Name of variable	Type	Length	Number of decimal figures	Not Null (Y/N)	Max value	Min value	Other constraints	Units	Description
QUE2TFFR	Number	1	0	N	7	1	-	-	On average, how often did you eat fresh fruit when you were a teenager? Q52<br>1 = Never<br>2 = Less than once a week<br>3 = 1-2 times a week<br>4 = 3-4 times a week<br>5 = 5-6 times a week<br>6 = Daily<br>7 = More than once a day
QUE2TBLS	Number	1	0	N	7	1	-	-	On average, how often did you eat blood sausage or liver sausage when you were a teenager? Q53<br>1 = Never<br>2 = Less than once a week<br>3 = 1-2 times a week<br>4 = 3-4 times a week<br>5 = 5-6 times a week<br>6 = Daily<br>7 = More than once a day
QUE2TVGT	Number	1	0	N	7	1	-	-	On average, how often did you eat vegetables when you were a teenager? Q54<br>1 = Never<br>2 = Less than once a week<br>3 = 1-2 times a week<br>4 = 3-4 times a week<br>5 = 5-6 times a week<br>6 = Daily<br>7 = More than once a day

Name of variable	Type	Length	Number of decimal figures	Not Null (Y/N)	Max value	Min value	Other constraints	Units	Description
QUE2TSMT	Number	1	0	N	6	1	-	-	On average, how often did you eat corned meat, corned meat sausage or another kind of salted/smoked meat when you were a teenager? Q55<br>1 = Never<br>2 = Less than once a month<br>3 = 1-3 times a month<br>4 = 1-2 times a week<br>5 = 3-6 times a week<br>6 = Daily or more often
QUE2TSFS	Number	1	0	N	6	1	-	-	On average, how often did you eat salt fish or smoked fish when you were a teenager? Q56<br>1 = Never<br>2 = Less than once a month<br>3 = 1-3 times a month<br>4 = 1-2 times a week<br>5 = 3-6 times a week<br>6 = Daily or more often
QUE2TRYB	Number	1	0	N	7	1	-	-	On average, how often did you eat rye bread or flatbread when you were a teenager? Q57<br>1 = Never<br>2 = Less than once a week<br>3 = 1-2 times a week<br>4 = 3-4 times a week<br>5 = 5-6 times a week<br>6 = Daily<br>7 = More than once a day

Name of variable	Type	Length	Number of decimal figures	Not Null (Y/N)	Max value	Min value	Other constraints	Units	Description
QUE2TOAT	Number	1	0	N	7	1	-	-	On average, how often did you eat oatmeal when you were a teenager? Q58<br>1 = Never<br>2 = Less than once a week<br>3 = 1-2 times a week<br>4 = 3-4 times a week<br>5 = 5-6 times a week<br>6 = Daily<br>7 = More than once a day
QUE2TCLO	Number	1	0	N	6	1	-	-	On average, how often did you take cod or saithe liver oil or liver oil pills (not halibut liver oil pills)when you were a teenager? Q59<br>1 = Never<br>2 = Less than once a week<br>3 = 1-2 times a week<br>4 = 3-4 times a week<br>5 = 5-6 times a week<br>6 = Daily
QUE2TMPR	Number	1	0	N	7	1	-	-	On average, how often did you drink milk or eat milk products when you were a teenager? Q60<br>1 = Never<br>2 = Less than once a week<br>3 = 1-2 times a week<br>4 = 3-4 times a week<br>5 = 5-6 times a week<br>6 = Daily<br>7 = More than once a day

Name of variable	Type	Length	Number of decimal figures	Not Null (Y/N)	Max value	Min value	Other constraints	Units	Description
QUE2TTOM	Number	1	0	N	5	1	-	-	What kind of milk did you most often get when you were a teenager? Q61<br>1 = Whole milk<br>2 = Skim milk<br>3 = Whey<br>4 = Cream/cream blend<br>5 = Never used milk
QUE2TSPR	Number	1	0	N	5	1	-	-	What kind of spread did you most often get when you were a teenager on bread or with meal? Q62<br>1 = Margarine<br>2 = Butter<br>3 = Tallow-cod liver oil blend<br>4 = Tallow/suet<br>5 = Didn't use spread
QUE2TBTR	Number	1	0	N	4	1	-	-	How did you generally butter your bread when you were a teenager? (Pictures) Q63<br>1 = Little butter<br>2 = Medium butter<br>3 = Much butter<br>4 = Usually didn't butter bread
QUE2RICK	Number	1	0	N	8	1			Do you know whether you got rickets as a child? Q64<br>0 = No<br>1 = Yes<br>8 = Don't know
QUE2ENGH	Number	1	0	N	4	1	-	-	Did you always get enough to eat when you were growing up? Q65<br>1 = Always got more then enough<br>2 = Got enough, but nothing more than that<br>3 = Sometimes didn't get enough<br>4 = Was often hungry as a child
